# Profilin modulates sarcomeric organization and mediates cardiomyocyte hypertrophy

**DOI:** 10.1093/cvr/cvw050

**Published:** 2016-03-07

**Authors:** Viola Kooij, Meera C. Viswanathan, Dong I. Lee, Peter P. Rainer, William Schmidt, William A. Kronert, Sian E. Harding, David A. Kass, Sanford I. Bernstein, Jennifer E. Van Eyk, Anthony Cammarato

**Affiliations:** 1 Department of Medicine, Division of Cardiology, The Johns Hopkins University, Baltimore, MD, USA; 2 National Heart and Lung Institute, Imperial College London, 4th floor, ICTEM, Hammersmith Campus, Du Cane Road, London W12 0NN, UK; 3 Division of Cardiology, Medical University of Graz, Graz, Austria; 4 Department of Biology, San Diego State University, San Diego, CA, USA; 5 Advanced Clinical Biosystems Research Institute, Heart Institute and Department of Medicine, Cedars-Sinai Medical Center, Los Angeles, CA, USA

**Keywords:** Profilin-1, Cardiac hypertrophy, Cardiomyocyte, Sarcomere remodelling, *chickadee*

## Abstract

**Aims:**

Heart failure is often preceded by cardiac hypertrophy, which is characterized by increased cell size, altered protein abundance, and actin cytoskeletal reorganization. Profilin is a well-conserved, ubiquitously expressed, multifunctional actin-binding protein, and its role in cardiomyocytes is largely unknown. Given its involvement in vascular hypertrophy, we aimed to test the hypothesis that profilin-1 is a key mediator of cardiomyocyte-specific hypertrophic remodelling.

**Methods and results:**

Profilin-1 was elevated in multiple mouse models of hypertrophy, and a cardiomyocyte-specific increase of profilin in *Drosophila* resulted in significantly larger heart tube dimensions. Moreover, adenovirus-mediated overexpression of profilin-1 in neonatal rat ventricular myocytes (NRVMs) induced a hypertrophic response, measured by increased myocyte size and gene expression. Profilin-1 silencing suppressed the response in NRVMs stimulated with phenylephrine or endothelin-1. Mechanistically, we found that profilin-1 regulates hypertrophy, in part, through activation of the ERK1/2 signalling cascade. Confocal microscopy showed that profilin localized to the Z-line of *Drosophila* myofibrils under normal conditions and accumulated near the M-line when overexpressed. Elevated profilin levels resulted in elongated sarcomeres, myofibrillar disorganization, and sarcomeric disarray, which correlated with impaired muscle function.

**Conclusion:**

Our results identify novel roles for profilin as an important mediator of cardiomyocyte hypertrophy. We show that overexpression of profilin is sufficient to induce cardiomyocyte hypertrophy and sarcomeric remodelling, and silencing of profilin attenuates the hypertrophic response.

## 1. Introduction

Heart failure (HF), a leading cause of morbidity and mortality, is often preceded by cardiac hypertrophy, a process in which cardiomyocytes exhibit increased size, changes in protein abundance, and cytoskeletal and sarcomeric reorganization.^1^ While current treatments offer therapeutic benefits, a greater understanding of the pathological underpinnings might enable more targeted modalities and improve survival. Thus, understanding the mediators of hypertrophy remains important.

Profilins are ubiquitously expressed, multifunctional, and highly conserved actin-binding proteins of ∼15 kDa.^2,3^ Four profilin genes have been identified in mammals, *Pfn1–Pfn4*,^4^ while the gene family in invertebrates is often less complex. In *Drosophila*, for example, a single profilin isoform is encoded by *chickadee.*^5^ Profilins are found in different cellular locations where they perform diverse cytoplasmic and nuclear roles.^4^ *Pfn1* encodes profilin-1, the isoform found in vertebrate cardiac tissue.^6^ It promotes actin polymerization by catalyzing ADP to ATP exchange on G-actin^3,7^ and through transient interactions of the profilin–ATP–actin complex with the fast-growing ‘barbed’ end of F-actin.^8^ Profilin associates with many ligands via its poly-l-proline-binding domain, linking it not only to proteins involved with actin polymerization, but also to Rac and Rho effector molecules, nuclear export receptors, and regulators of endocytosis.^3^ Profilin also interacts with phosphatidylinositol lipids^6,9,10^ and transcription factors, highlighting roles in signalling and gene activity.^11^ Its promiscuous associations with numerous ligands underscores profilin's connection to several diseases including familial amyotrophic lateral sclerosis^12^ and to common hypertrophic signalling pathways.^13^

Myofibrils of cardiac and skeletal myocytes are highly differentiated cytoskeletal structures, in which F-actin and other contractile components are tightly organized into individual, repetitive sarcomeric units.^14^ Within each sarcomere, F-actin-containing thin filaments have their barbed ends anchored at the Z-line, whereas the slow-growing ‘pointed’ ends extend towards the centrally located M-line.^15^ Myofibrils are straight and uniform in length,^16^ but can change upon pathological stimuli, whereby sarcomeres are added in series. The significance in striated muscle maintenance of actin-binding proteins that regulate different aspects of F-actin formation and stability has been reported for cofilin-2,^17^ Wdr1,^18^ leiomodin-2,^19^ and gelsolin.^20^ Importantly, the latter, an actin severing and capping protein, has recently be shown to regulate cardiac remodelling following myocardial infarction.^20^

It has been established that profilin-1 plays key roles in smooth muscle contraction^21^ and vascular remodelling.^13^ Increased expression of profilin-1 in vascular smooth muscle cells induced stress fibre formation, triggered ERK1/2, JNK, and Rho-associated hypertrophic signalling cascades, and resulted in elevated blood pressure.^13^ Profilin-1 is highly expressed in left ventricles of spontaneously hypertensive rats (SHRs) and promotes cardiac hypertrophy and fibrosis by modulating actin polymerization.^22^ Nevertheless, it is unclear whether this is a primary consequence of altered profilin-1 in cardiomyocytes, or a secondary effect in response to changes in profilin-1 in the vasculature, as evidenced by Elnakish *et al.*^23^ who demonstrated that vascular remodelling-associated hypertension engendered left ventricular hypertrophy and contractile dysfunction in transgenic mice overexpressing profilin-1. Thus, we tested the hypothesis that cardiomyocyte hypertrophy is accompanied by altered profilin-1 levels and that such muscle-specific changes, *in vivo*, can directly modulate sarcomere organization and independently drive cellular remodelling. Our results reveal key roles for profilin-1 as a mediator of cardiomyocyte hypertrophy, as a regulator of myofibrillar and sarcomeric organization, and as a key signalling molecule that is both necessary and sufficient for cellular remodelling.

## 2. Methods

Expanded methods are available in Supplementary material online.

### 2.1 Animal models and fly strains

Sham-operated male C57BL/6 mice (8–11 weeks, Jackson Laboratories, Bar Harbor, ME, USA) or mice with induced pressure-overload of the left ventricle via transverse aortic constriction (TAC) were investigated.^24^ TAC was performed by tying a suture (7-0 prolene) around the transverse aorta and a 26-gauge needle. In addition, Gαq-overexpressing FNB/N male mice (4–5 months) and non-transgenic controls were used.^25^ Details on anaesthesia, analgesia, and euthanasia are described in Supplementary material online. Protocols were approved by the Johns Hopkins Medical Institutions Animal Care and Use Committee, and the animal experiments that were performed conform to the NIH guidelines.

Flies were raised and crossed at 25°C according to standard procedures. ‘Profilin-1’ denotes the mammalian isoform and ‘profilin’ the *Drosophila* homologue. The following fly stocks were used: *w; CyO;P*[*UAS* + *chicE1*] *78.3* (UAS-Pfn_1) and *w; CyO;P*[*UAS* + *chicE1*]*36.5* (UAS-Pfn_2) (kind gifts of Dr Lynn Cooley, Yale University),^26^ *γw; Dmef2-GAL4* (Bloomington Stock Center), *w, UH3-GAL4* (kind gift of Dr Upendra Nongthomba, Indian Institute of Science),^27^ *w; Hand-GAL4* (Bloomington Stock Center), *γν; UAS-Chic^RNAi^* (*y*^1^ sc* *v*^1^; *P{TRiP.HMS00550}attP2*, Bloomington Stock Center).

The GAL4-upstream activator sequence (UAS) system was utilized for targeted *Drosophila* gene expression, in which the yeast GAL4 transcription factor activates transcription of its target genes by binding to UAS *cis*-regulatory sites.^28^ The combination of two transgenic fly lines (UAS-Pfn_1 and UAS-Pfn_2) with two muscle driver lines (Mef2-GAL4 and UH3-GAL4) created a genotypically diverse range of profilin overexpression and content among offspring. Progeny of *w^1118^* or *γw* flies crossed with the appropriate driver line served as controls. Adult flies were used for all experiments. Ten hours after emerging from puparia, adult female flies are sexually mature, begin to breed, and lay eggs.

### 2.2 Western blot analysis

Western blot analysis was done according to standard protocols. Western blots of tissue from mice and *Drosophila* were corrected for loading using Direct Blue 71- or Pierce Reversible Protein Stain-stained membranes. For this purpose, intensity over the entire lane was averaged.

### 2.3 Viral transfection, RNA interference, and RNA isolation from neonatal rat ventricular myocytes

Neonatal rat ventricular myocytes (NRVMs) were Isolated and then cultured from 1- to 2-day-old Sprague–Dawley rats as previously described.^29^ Overexpression of profilin-1 was achieved via adenovirus-mediated transfection. Ad-mCherry-mPFN1 and Ad-mCherry (Adenoviral Type 5, CMV promoter) were purchased from Vector Biolaboratories (Burlingame, CA, USA). NRVMs were transfected using a multiplicity of infection of 10 for 24 h. RNA was harvested 24 h after and protein 48 or 72 h after transfection. For RNA interference, ON-TARGET and SMART pool reagent against *Pfn1* (L-092311-02) were purchased from Dharmacon (Lafayette, CO, USA). ON-TARGET and Non-targeting pool (D-001810-10-05, Dharmacon) were used as a non-specific control. Twenty-four hours after plating, NRVMs were transfected with 25 nM siRNA using DharmaFECT 1 (Dharmacon) following the manufacturer's protocol. The next day, cells were treated with 20 μM phenylephrine (PE) or 100 nM endothelin-1 (ET1). RNA isolation is described in detail in Supplementary materials online.

### 2.4 Confocal microscopy and EM

Mouse myocardium was fixed with 10% formalin, paraffin-embedded, and sectioned into 4 μm slices. Indirect flight muscles (IFMs) from 2- to 3-day-old adult flies were dissected from bisected half thoraces and fixed in 4% paraformaldehyde overnight. Samples were labelled for confocal microscopy according to standard techniques.^24,30^ Composite averaged confocal images of consecutive *Drosophila* IFM sarcomeres were created using a novel ImageJ-based approach. EM of IFM was conducted as reported previously.^31^ Complete details regarding sample processing, staining, and imaging procedures can be found in Supplementary material online.

### 2.5 *Drosophila* flight and climbing tests, and image analysis of beating hearts

Flight and climbing tests were carried out on 2–3-day-old adult flies. Cardiac tubes of 3-week-old female adult flies were surgically exposed according to Vogler and Ocorr^32^ High-speed movies of semi-intact *Drosophila* preparations were acquired for image analysis of heart contractions as previously described.^30,33^ Thirty-second movies were taken at ∼120 frames per second using a Hamamatsu Orca Flash 2.8 CMOS camera on a Leica DM5000B TL microscope with a ×10 immersion lens. M-mode kymograms were generated, and physiological parameters assessed, using a MATLAB-based image analysis program.^34^

### 2.6 Statistics

Prism 5 (GraphPad Software) was used for statistical analyses and graphical presentations. Statistical tests employed are described in figure legends.

## 3. Results

### 3.1 Mammalian hypertrophic hearts are characterized by increased profilin-1 content

**Figure 1:**
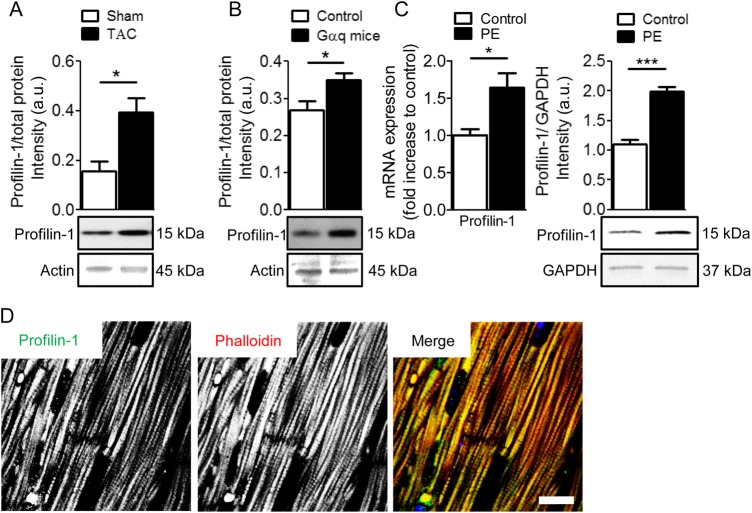
Increased expression of profilin-1 in hypertrophic cardiomyocytes. (*A*) Pressure-overload following TAC resulted in elevated levels of profilin-1 in mouse hearts. Protein levels were corrected for total gel loading (see Supplementary material online, *Figure S1A*). Representative actin bands from Direct Blue 71-stained membranes are shown. A significant increase in profilin-1/total protein was observed in the myocardium of the TAC vs. Sham group (*n* = 5–10, *P < 0.05; Student's *t*-test). (*B*) A hypertrophic/HF mouse model overexpressing wild-type Gαq showed higher levels of cardiac profilin-1 compared with control (*n* = 3–7, *P < 0.05; Student's *t*-test). Protein levels were corrected for total gel loading (see Supplementary material online, *Figure S1A*). Representative actin bands from Direct Blue 71-stained membranes are shown. (*C*) PE treatment in NRVMs increased *Pfn1* mRNA (*n* = 6, *P < 0.05; Student's *t*-test) and profilin-1 (*n* = 6, ***P < 0.001; Student's *t*-test) content. (*D*) Representative confocal images of control murine cardiac tissue show profilin-1 repetitively associates with myofibrils in a striated manner. Scale bar, 10 μm.

To determine whether profilin-1 abundance in the heart is altered in different animal models of cardiac hypertrophy and HF, western blot analysis was performed on ventricular tissues from mice that underwent TAC (*Figure 1A* and see Supplementary material online, *Figure S1A*)^35^ and from Gαq-overexpressing mice and appropriate controls (*Figure 1B* and see Supplementary material online, *Figure S1*A).^25^ Relative to total protein, profilin-1 levels were ∼2.5-fold higher in the TAC group (0.40 ± 0.06 a.u., *n* = 10) compared with the control group (0.16 ± 0.04 a.u., *n* = 5). TAC animals additionally demonstrated cardiac dysfunction (see Supplementary material online, *Figure S1B*). Moreover, cardiac tissue obtained from Gαq-overexpressing mice showed significantly increased levels of profilin-1 (0.35 ± 0.02 a.u., *n* = 7) compared with controls (0.27 ± 0.02 a.u., *n* = 3). NRVMs were isolated to assess cardiomyocyte-specific expression levels of *Pfn1* (profilin-1) in cells treated with PE or ET1 to stimulate hypertrophy. *Pfn1* transcripts were significantly increased after stimulation with 20 μM PE for 24 h (1.7 ± 0.22, *n* = 6) compared with unstimulated NRVMs (1.0 ± 0.05, *n* = 6; *Figure 1C*), and also in NRVMs treated with 100 nM ET1 (1.3 ± 0.12, *n* = 6) relative to controls (1.0 ± 0.08, *n* = 6; see Supplementary material online, *Figure S1C*). Furthermore, profilin-1 was significantly more abundant after PE treatment (2.0 ± 0.08, *n* = 6) compared with untreated controls (1.1 ± 0.08, *n* = 6). To define the gross localization of profilin-1, sectioned cardiac tissue from control mice was subjected to anti-profilin-1 antibody, DAPI, and TRITC-phalloidin staining. Confocal images showed a striated profilin-1 signal, which implies the protein associates recurrently along sarcomeres (*Figure 1D*). This is consistent with earlier results,^6^ confirming the presence of profilin-1 in cardiomyocytes, and repetitive occupancy of profilin-1 along myofibrils. Cardiac tissues from explanted hearts of patients with end-stage HF (Failing) contained decreased *PFN1* transcript levels (0.55 ± 0.04 a.u. *n* = 9) compared with donor hearts (Healthy, 1.05 ± 0.21 a.u., *n* = 8; see Supplementary material online, *Table S1* and *Figure S1D*). The discrepancy in profilin-1/*Pfn-1*/*PFN1* levels between the hypertrophic hearts and human end-stage failing hearts may be due to differences in disease and diseased (e.g. compensated vs. decompensated) state.

### 3.2 Cardiomyocyte-specific overexpression of profilin induces cardiomyopathy in *Drosophila*

**Figure 2:**
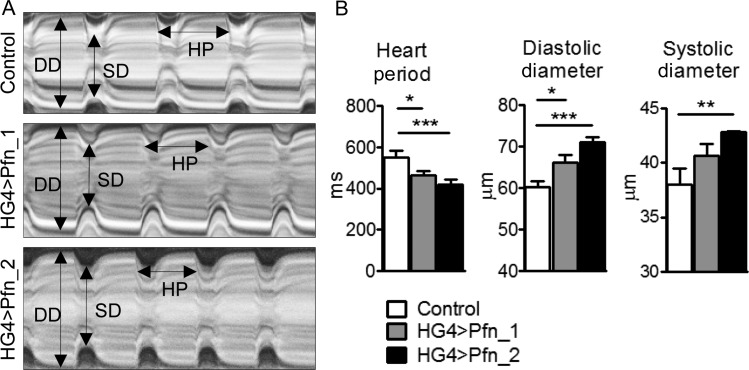
Cardiomyocyte-specific overexpression of profilin in *Drosophila* induces cardiomyopathy. (*A*) Representative M-mode kymograms generated from high-speed videos of beating control, Pfn_1, and Pfn_2 heart tubes. DD, diastolic diameter; SD, systolic diameter; HP, heart period. (*B*) Semi-automated optical heartbeat analysis from flies overexpressing profilin via the HG4 cardiac-specific driver revealed significant reductions in heart period and increased cardiac dimensions relative to control (*n* = 28–30, *P < 0.05, **P < 0.01 and ***P < 0.001; Kruskal–Wallis test with Dunn's *post hoc* test for HP and SD analysis; one-way ANOVA with the Bonferroni *post hoc* test for DD analysis).

To investigate cardiomyocyte-restricted effects of increased profilin expression *in vivo*, and to assess whether elevated profilin quantity is sufficient to alter contractile performance and/or cardiac dimensions in a tissue-specific manner, two independent transgenic fly lines (UAS-Pfn_1 and UAS-Pfn_2) were crossed with flies harbouring the heart-specific Hand-GAL4 (HG4) driver (*Figure 2A*). Cardiac-restricted overexpression of profilin in the progeny resulted in significantly reduced heart periods (HG4 > Pfn_1 464 ± 23 ms, *n* = 31; HG4 > Pfn_2 421 ± 21 ms, *n* = 30), which indicated increased heart rate, compared with control (553 ± 30 ms, *n* = 28; *Figure 2B*). Diastolic diameters were significantly enlarged in HG4 > Pfn_1 (66 ± 2 μm, *n* = 31) and HG4 > Pfn_2 (71 ± 1 μm, *n* = 30) relative to control (60 ± 1 μm, *n* = 28) flies, as were systolic diameters in HG4 > Pfn_2 (43 ± 1 μm, *n* = 30) compared with control (38 ± 2 μm, *n* = 28; *Figure 2B*). Knockdown of profilin in cardiomyocytes (HG4 > Pfn^RNAi^) was maladaptive and resulted in lethality, as flies did not eclose from their puparia. These data suggest that profilin is essential for adult *Drosophila* cardiac development, and that its overexpression induces a phenotype reminiscent of eccentric hypertrophy.^36^

### 3.3 Myocyte-specific overexpression of profilin impairs muscle function and ultrastructure

**Figure 3:**
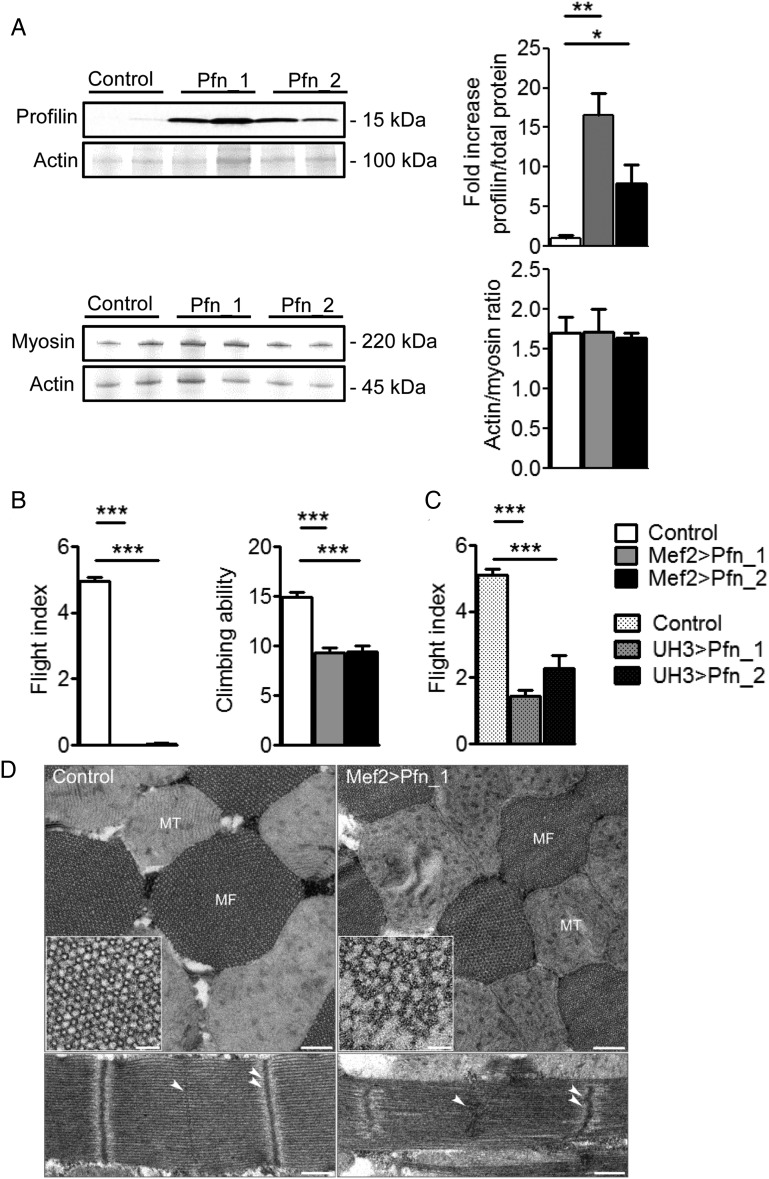
Overexpression of profilin in *Drosophila* IFM impairs muscle function and ultrastructure. (*A*) Western blot analysis showed increased profilin in whole Mef2 > Pfn_1 and Mef2 > Pfn_2 transgenic flies (top) (*n* = 5, *P < 0.05, **P < 0.01; one-way ANOVA with the Bonferroni *post hoc* test). Actin/myosin heavy chain ratios remained unchanged in flies with muscle-restricted profilin overexpression compared with control (bottom) (*n* = 5). (*B*) Two-day-old Mef2 > Pfn_1 and Mef2 > Pfn_2 flies were unable to fly and demonstrated significantly reduced climbing ability (*n* = 35–64, ***P < 0.001; Kruskal–Wallis test with Dunn's *post hoc* test). (*C*) UH3-GAL4-mediated overexpression of profilin significantly diminished flight ability (*n* = 54–83, ***P < 0.001; one-way ANOVA with the Bonferroni *post hoc* test). (*D*) Representative electron micrographs of transverse sections of Mef2 > Pfn_1 IFMs (top) show that the double hexagonal lattice of myofilament arrangement was less ordered and thin and thick filaments were missing on the outer edges of the myofibril (inset) relative to control. Moreover, there was Z-band buckling and M-line distortion in longitudinal sections (bottom). Single arrowheads delineate an M-line and double arrowheads a Z-line. MT, mitochondrion; MF, myofibril. Scale bars, 500 nm and 250 nm for longitudinal and transverse sections, respectively, and 50 nm in the inset.

To further index myopathic effects associated with elevated profilin levels, transgenic *Drosophila* overexpressing Pfn_1 and Pfn_2 throughout the somatic musculature were established using the Mef2-GAL4 driver line. Mef2 > Pfn_1 flies (*n* = 5) exhibited a significant, ∼17-fold increase of profilin, whereas Mef2 > Pfn_2 flies (*n* = 5) showed an ∼8-fold increase compared with controls (*Figure 3A*). Actin/myosin heavy chain (MHC) ratio and individual intensity values normalized to total intensity, determined by densitometric analysis of Coomassie-stained protein bands, were not altered in these flies (*Figure 3A* and see Supplementary material online, *Figure S2*). Muscle performance was evaluated in 2-day-old flies using flight and climbing assays. Elevated profilin eliminated flight and reduced climbing abilities (control 14.91 ± 0.50 cm, *n* = 64; Mef2 > Pfn_1 9.29 ± 0.56 cm, *n* = 48; Mef2 > Pfn_2 9.43 ± 0.58 cm, *n* = 35; *Figure 3B*). Furthermore, IFM-specific overexpression of profilin via UH3-GAL4^27^ reduced, but did not completely abolish flight ability compared with control animals (control 5.11 ± 0.17 a.u., *n* = 83; UH3 > Pfn_1 1.43 ± 0.19 a.u., *n* = 74; UH3 > Pfn_2 2.30 ± 0.39 a.u., *n* = 54; *Figure 3C*).

Due to an extremely well-organized myofilamentous lattice, *Drosophila* IFM myofibrils are highly amenable to structural analysis. To ascertain if profilin overexpression produced ultrastructural abnormalities, we examined IFMs of 2-day-old control and Mef2 > Pfn_1 flies by transmission EM (*Figure 3D*). Transverse sections through the IFM revealed that the double hexagonal lattice of thick and thin filaments in Mef2 > Pfn_1 flies was disorganized relative to control. Closer examination revealed filament loss around the periphery of the Mef2 > Pfn_1 myofibrils (inset), which was not observed in control flies. Moreover, elevated profilin perturbed sarcomeric Z- and M-line appearance and increased sarcomere lengths (control 2.75 ± 0.04 μm, *n* = 20; Mef2 > Pfn_1 3.00 ± 0.03 μm, *n* = 20). Reduced profilin expression using either the Mef2-GAL4 or UH3-GAL4 driver lines in conjunction with UAS-Pfn^RNAi^ prevented adult *Drosophila* emergence from their respective pupal cases. This underscores the fundamental importance of profilin for muscle development.

### 3.4 Elevated profilin results in elongated thin filaments and sarcomeres and its accumulation at the centre of the sarcomere

**Figure 4:**
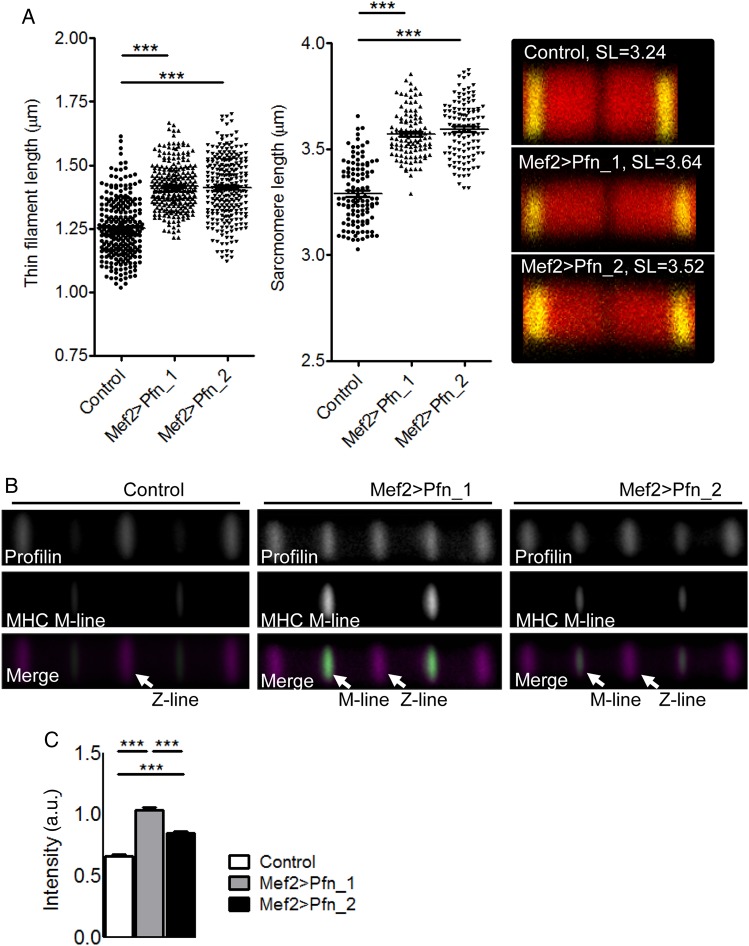
Elongated thin filaments and sarcomeres in flies overexpressing profilin. (*A*) Left: increased IFM thin filament (*n* = 252–255, ***P < 0.001; one-way ANOVA with the Bonferroni *post hoc* test) and sarcomere lengths (*n* = 106–116, ***P < 0.001; one-way ANOVA with the Bonferroni *post hoc* test) were measured in flies with Mef2-GAL4-driven profilin overexpression. Right: typical IFM sarcomeres for control and profilin-overexpressing flies (red, TRITC-phalloidin-stained thin filaments; yellow, anti-α-actinin-stained Z-lines). (*B*) Averaged composite images of consecutive IFM sarcomeres from flies with elevated profilin levels revealed localization at the Z-line and at the thin filament pointed end/H-zone, whereas controls predominantly showed profilin localization at the Z-line. The M-line/H-zone was labelled using an MHC antibody that recognizes the centre of thick filaments along IFM myofibrils. (*C*) Based on normalized fluorescence intensity, Mef2 > Pfn_1 and Mef2 > Pfn_2 transgenic flies had significantly more profilin at the M-lines/H-zones, proximal to the thin filament pointed ends, relative to that at the Z-lines compared with controls (*n* = 123–143, ***P < 0.001; one-way ANOVA with the Bonferroni *post hoc* test).

To verify an effect of muscle-restricted profilin overexpression on thin filament and sarcomere lengths, *Drosophila* IFM myofibrils were labelled with anti-α-actinin antibody, a Z-line protein, and TRITC-phalloidin, imaged via confocal microscopy, and dimensions ascertained (see Supplementary material online, *Figure S3A*). Increased expression of profilin via the Mef2-GAL4 driver (*Figure 3A*) resulted in significantly elongated thin filament (Mef2 > Pfn_1 1.42 ± 0.01 μm, *n* = 255; Mef2 > Pfn_2 1.41 ± 0.01 μm, *n* = 252) and sarcomere lengths (Mef2 > Pfn_1 3.57 ± 0.01 μm, *n* = 106; Mef2 > Pfn_2 3.59 ± 0.01 μm, *n* = 116) compared with control thin filament (1.25 ± 0.01 μm, *n* = 255) and sarcomere lengths (3.29 ± 0.01 μm, *n* = 114; *Figure 4A*). These results are consistent with significantly increased sarcomere lengths measured from electron micrographs (*Figure 3D*) and were confirmed in flies overexpressing profilin in the IFM using the UH3-GAL4 driver line (thin filament: control 1.28 ± 0.01 μm, *n* = 274; UH3 > Pfn_1 1.46 ± 0.01 μm, *n* = 274; UH3 > Pfn_2 1.42 ± 0.01 μm, *n* = 271; sarcomere lengths: control 3.31 ± 0.01 μm, *n* = 274; UH3 > Pfn_1 3.61 ± 0.01 μm, *n* = 98; UH3 > Pfn_2 3.63 ± 0.01 μm, *n* = 104; see Supplementary material online, *Figure S3B*).

To resolve the sarcomeric localization of profilin, half thoraces from control flies were labelled with TRITC-phalloidin, anti-profilin antibody, and with an anti-MHC antibody that labels the H-zone/M-line region of IFM myofibrils. Confocal images of myofibrils demonstrated that profilin localized predominantly to the Z-line of the sarcomere under basal conditions, a position expected due to its known association with the barbed end of F-actin (*Figure 4B*). Myofibrils from both Mef2 > Pfn_1 and Mef2 > Pfn_2 overexpressors, however, showed profilin at the Z-line and a significant accumulation at a position towards the thin filament pointed end/H-zone (*Figure 4B*). The average of the ratios of the maximum profilin intensity at each pointed end/H-zone to the maximum profilin intensity at the neighbouring Z-line was significantly higher for Mef2 > Pfn_1 (1.03 ± 0.02, *n* = 123) and Mef2 > Pfn_2 (0.85 ± 0.02, *n* = 143) myofibrils compared with control (0.66 ± 0.02, *n* = 138; *Figure 4C*). In summary, data generated using the *Drosophila* model illustrate that myocyte-restricted overexpression of profilin is sufficient to induce sarcomeric and myofibrillar remodelling, muscle dysfunction, and myopathy.

### 3.5 Adenoviral overexpression of profilin-1 induces a hypertrophic response in NRVMs

**Figure 5:**
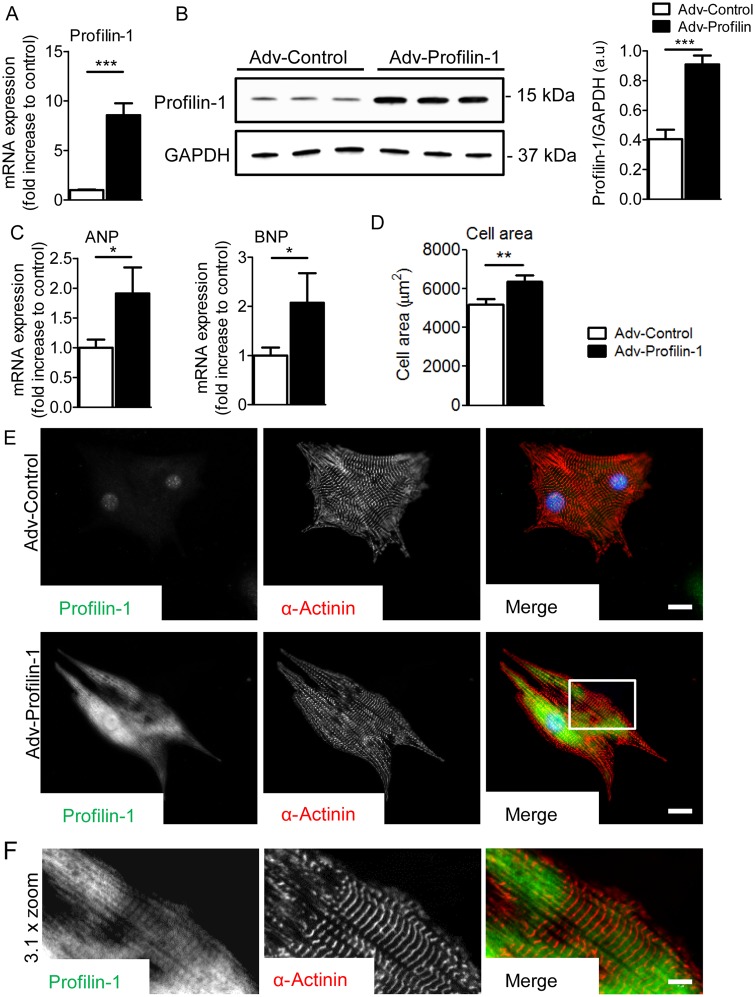
Adenoviral-mediated overexpression of profilin-1 induces a hypertrophic response in NRVMs. (*A*) Transcript levels of *Pfn1* in NRVMs were significantly higher than control following adenoviral-mediated transfection (*n* = 12, ***P < 0.001; Student's *t*-test). (*B*) *Pfn1* overexpression resulted in significantly increased levels of profilin-1 (*n* = 6, ***P < 0.001; Student's *t*-test). (*C*) Elevated *Pfn1* expression resulted in increased transcript levels of the hypertrophic markers ANP and BNP (*n* = 12, *P < 0.05; Student's *t*-test). (*D*) NRVMs exhibited significantly larger cellular areas in response to *Pfn1* overexpression (*n* = 33, **P < 0.01; Student's *t*-test). (*E*) Representative profilin-1 and α-actinin antibody-stained confocal images of Adv-Control- and Adv-Profilin-1-transfected NRVMs. Scale bar, 15 µm. (*F*) A ×3.1 zoom of confocal images of Adv-Profilin-1-transfected cells (white box in the merged image). Scale bar, 5 µm.

Adenoviral-mediated profilin-1 overexpression in NRVMs resulted in significant increases in *Pfn1* mRNA (*n* = 12) and protein (*n* = 6) levels (*Figure 5A* and *B*) compared with controls (Adv-Control). Increased levels of profilin-1 resulted in elevation of the hypertrophic fetal gene markers atrial natriuretic peptide (ANP) and brain natriuretic peptide (BNP) (*Figure 5C*). Additionally, increased cell size, another hallmark of hypertrophy, was measured in cells transfected with Adv-Profilin-1 (6387 ± 317 µm^2^, *n* = 33) compared with Adv-Control (5207 ± 260 µm^2^, *n* = 33; *Figure 5D*). Both adenoviruses expressed mCherry under the control of the CMV promoter to determine transfection efficiency. *Figure 5E* shows representative images of profilin-1 and α-actinin in NRVMs transfected with Adv-Profilin-1 and Adv-Control. Profilin-1 frequently exhibited repetitive occupancy along NRVM myofibrils (*Figure 5F*). These results indicate that overexpression of profilin-1 in NRVMs is sufficient to induce cellular hypertrophy.

### 3.6 Suppression of *Pfn1* gene expression attenuates hypertrophic signalling in NRVMs

**Figure 6:**
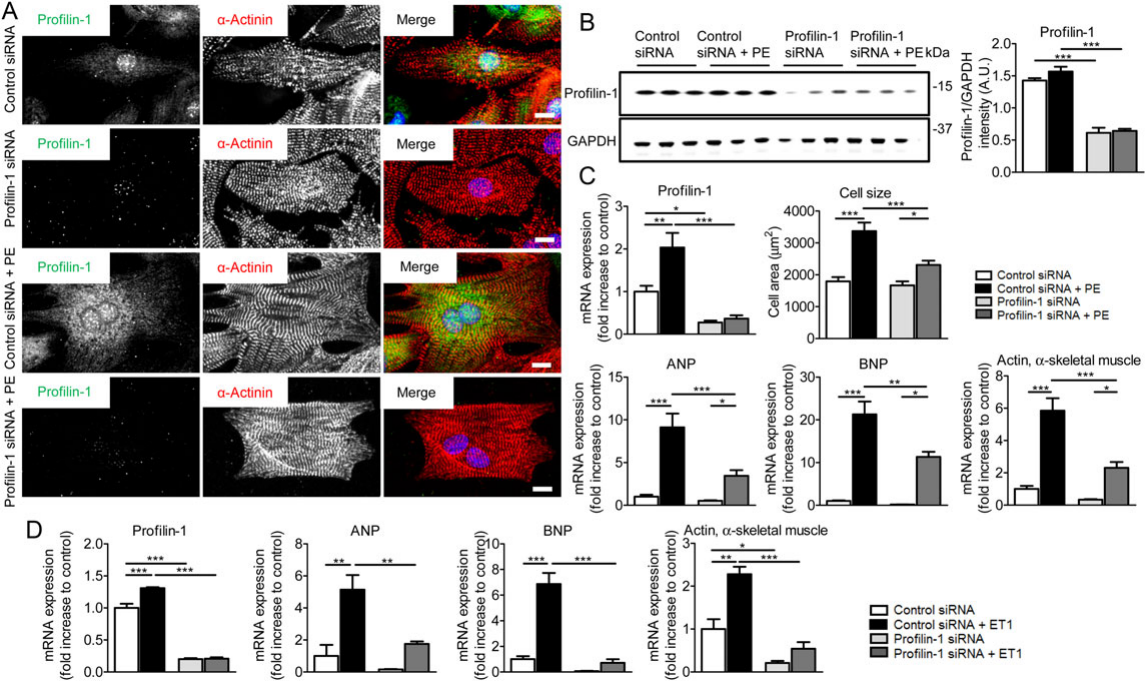
Silencing of *Pfn1* attenuates hypertrophic signalling in NRVMs. (*A*) Representative confocal images of control and PE-stimulated NRVMs. NRVMs were treated with control siRNA or *Pfn1* siRNA. Nuclei were stained with DAPI (blue). Profilin-1 was dramatically reduced in response to *Pfn1* siRNA. Scale bar, 10 μm. (*B*) Western blot analysis showed significantly decreased profilin-1 levels after the treatment of NRVMs with *Pfn1* siRNA (*n* = 3, ***P < 0.001; two-way ANOVA with the Bonferroni *post hoc* test). (*C*) Transcript levels of *Pfn1* in PE-stimulated NRVMs were increased compared with control, and treatment with *Pfn1* siRNA significantly reduced mRNA levels (*n* = 4, *P < 0.05, **P < 0.01, and ***P < 0.001; two-way ANOVA with the Bonferroni *post hoc* test). Cell surface area increased significantly upon treatment with PE and was diminished upon profilin-1 silencing (*n* = 20–29, *P < 0.05 and ***P < 0.001; two-way ANOVA with the Bonferroni *post hoc* test). Transcription of the hypertrophic markers ANP, BNP, and skeletal muscle actin was significantly reduced after *Pfn1* silencing in PE treated cells (*n* = 4, *P < 0.05, **P < 0.01, and ***P < 0.001; two-way ANOVA with the Bonferroni *post hoc* test). (*D*) NRVMs that were treated with *Pfn1* siRNA and stimulated with ET1 exhibited significantly reduced ANP, BNP, and skeletal α-actin mRNA levels compared with control siRNA-treated cells (*n* = 3, *P < 0.05, **P < 0.01, and ***P < 0.001; two-way ANOVA with the Bonferroni *post hoc* test).

Profilin-1 protein and mRNA levels are increased compared to appropriate controls following TAC, in Gαq-overexpressing mouse hearts, and in PE/ET1-stimulated NRVMs, respectively (*Figure 1A*–*C*). Likewise, when overexpressed exclusively in *Drosophila* cardiomyocytes, profilin promoted eccentric hypertrophy (*Figure 2*). To further test if increased profilin-1 is vital to the cardiac hypertrophic response, we expressed *Pfn1* siRNA in NRVMs and subsequently exposed the cells to PE or ET1. siRNA-directed *Pfn1* silencing was confirmed by confocal microscopy (*Figure 6A*) and western blot analysis (*Figure 6B*). In addition, reduced and elevated *Pfn1* mRNA levels verified the cellular responses to *Pfn1* siRNA and post PE treatment, respectively (*Figure 6C*). PE resulted in significantly larger cells (3372 ± 266 µm^2^, *n* = 20) compared with control (1790 ± 138 µm^2^, *n* = 26; *Figure 6C*). Myocytes treated with *Pfn1* siRNA and then PE had an increased surface area/size (2308 ± 135 µm^2^, *n* = 21) compared with unstimulated *Pfn1* siRNA cells (1668 ± 122 µm^2^, *n* = 29). However, they were significantly smaller than PE-stimulated controls (3372 ± 266 µm^2^, *n* = 20). Moreover, ANP, BNP, and skeletal muscle actin were significantly decreased in cells treated with *Pfn1* siRNA followed by PE stimulation compared with control cells. These findings were corroborated using ET1 stimulation of NRVMs in conjunction with *Pfn1* silencing (*Figure 6D*). Our results indicate that profilin-1 contributes to hypertrophy-induced cell growth.

**Figure 7:**
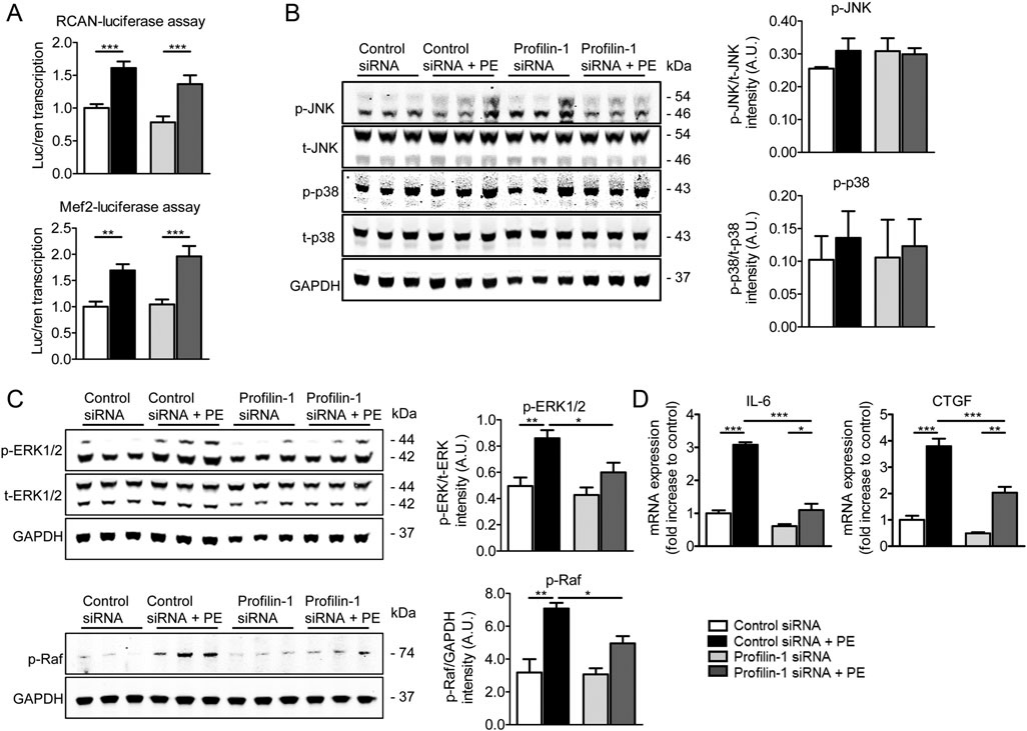
Profilin-1 is involved in the ERK1/2 signalling pathway. (*A*) The transcriptional activity of RCAN and MEF2 significantly increased when cells were stimulated with PE. Activity did not decrease upon silencing of profilin-1 (*n* = 5, **P < 0.01 and ***P < 0.001; two-way ANOVA with the Bonferroni *post hoc* test). (*B*) Phosphorylation levels of JNK (corrected for total JNK) and p38 (corrected for total p38) were unaltered by *Pfn1* silencing and PE treatment (*n* = 3, two-way ANOVA with the Bonferroni *post hoc* test). (*C*) Phosphorylation of ERK1/2 (corrected for total ERK1/2) and Raf (corrected for GAPDH) was significantly increased in hypertrophic NRVMs and reduced upon diminished profilin-1 expression (*n* = 3, *P < 0.05 and **P < 0.01; two-way ANOVA with the Bonferroni *post hoc* test). (*D*) PE-increased mRNA levels of IL-6 and CTGF, effector genes of the ERK1/2 signalling cascade, were significantly reduced upon silencing of *Pfn1* (*n* = 3, *P < 0.05, **P < 0.01, and ***P < 0.001; two-way ANOVA with the Bonferroni *post hoc* test).

To elucidate the signalling pathway involved in profilin-1-mediated cardiomyocyte-specific remodelling, we evaluated the activity of two major transcription factors (NFAT and MEF2) that govern the stress response during cardiac hypertrophy.^37^ Activity was measured using luciferase assays with the regulator of calcineurin (RCAN, an upstream regulator of NFAT) and MEF2 reporters. Following incubation with *Pfn1* siRNA and PE, RCAN and MEF2 luciferase signals were not reduced (*n* = 5), suggesting the profilin-1-associated hypertrophic response relies on alternative signal transduction pathways (*Figure 7A*). Next, involvement of the mitogen-activated protein kinase (MAPK) hypertrophic signalling pathway was tested. The amount of phosphorylated JNK and p38 was not significantly different among the groups and thus did not appear to be involved (*n* = 3; *Figure 7B*). However, activated (phosphorylated active sites) levels of ERK1/2 (Thr 202/Tyr 204) and Raf (Ser 338) were significantly reduced in cells treated with PE and *Pfn1* siRNA compared with control PE-treated cells, indicating that profilin-1 is likely involved in the ERK1/2 MAPK hypertrophic signalling pathway (*n* = 3; *Figure 7C*). Consistent with this result, we measured reduced transcript levels of two downstream genes, *IL-6* and *CTGF*, of the ERK1/2 signalling pathway upon silencing of *Pfn1* in cells stimulated with PE (*Figure 7D*) and ET1 (see Supplementary material online, *Figure S4*). Particular proteins, collagens, actin isoforms, and actin-binding proteins, however, remained unaltered following PE stimulation in cardiomyocytes with profilin-1 knockdown (see Supplementary material online, *Figure S5A–C*).

## 4. Discussion

Our results, obtained using a combination of diverse but complementary model systems, reveal key roles for profilin as a potent mediator of cardiomyocyte hypertrophy, as a regulator of myofibrillar and sarcomeric organization, and as a signalling molecule. Profilin-1 expression was increased in left ventricles of mice hearts with cardiac dysfunction. Consistent with this, cardiomyocyte-specific elevation of profilin in the *Drosophila* heart tube increased cardiac dimensions, and overexpression of profilin-1 in NRVMs induced a hypertrophic response. Mechanistically, we found that elevated expression resulted in elongated thin filaments and sarcomeres, led to dysfunctional and disrupted myofibrils in fly muscle and, that in vertebrate cardiomyocytes, profilin-1 regulated hypertrophy through activation of the ERK1/2 signalling cascade.

### 4.1 Cardiomyocyte-specific expression of profilin-1

Recently, Zhao *et al.*^22^ demonstrated that profilin-1 was highly expressed in left ventricles of hearts isolated from SHRs. The authors used adenovirus tail vein injections to knockdown or overexpress profilin-1 ubiquitously. Global knockdown of profilin-1 in SHRs attenuated cardiac hypertrophy, while overexpression promoted it. It remained unclear, however, whether hypertrophy was a response to cardiomyocyte-specific increases of profilin-1 and/or vascular-specific remodelling due to increased *Pfn1* expression in smooth muscle or endothelial cells. Our data extend these findings and confirm an increase of profilin-1 in two different mouse models of hypertrophy and HF, indicating that expression of profilin-1 is up-regulated two- to three-fold in cardiomyocytes independent of disease stimuli. We additionally observed a cardiomyocyte-specific increase in *Pfn1* mRNA in cell models of hypertrophy, and overexpression of profilin-1 was sufficient to induce a hypertrophic response. Thus, our data verify that elevated levels of profilin-1 in failing hearts are, at least in part, due to cardiomyocyte-restricted expression changes. We cannot conclude, however, whether increased profilin levels *in vivo* are causal or secondary to cardiac dysfunction.

### 4.2 Altered sarcomeric structure due to profilin overexpression

To discern sub-sarcomeric localization of profilin, we imaged *Drosophila* IFM myofibrils. *Drosophila* IFM comprises extremely well-organized myofibrils that are comparable to those found in myocardium. IFM function can easily be tested by evaluating flight ability, and because of the highly organized fibrillar nature of the muscle, defects in myofibrillar and sarcomeric organization are readily observable. Experiments were performed using two transgenic fly lines (UAS-Pfn_1 and UAS-Pfn_2) with two muscle driver lines (Mef2-GAL4 and UH3-GAL4) to obtain a range of muscle-restricted profilin overexpression levels. This helped distinguish profilin-induced effects from potential non-specific actions. Since all genotypic combinations of fly strains demonstrated similarly afflicted myocytes, profilin apparently promotes distinct alterations to myofibrillar function and structure regardless of the level of overexpression. However, we cannot completely exclude the possibility that excessively high overexpression, using the Mef2-Gal4 driver, may have introduced imperceptible, non-specific events. We detected profilin localized predominantly to the Z-line in adult control sarcomeres and to the Z- and the pointed end/M-line region when overexpressed. Moreover, elevated levels of profilin resulted in elongated sarcomeres and thin filaments. Similar results were obtained by Bai *et al.*^38^ for the actin-binding, WH2-domain-containing protein sarcomere length short (SALS), indicating it too is required for proper sarcomere length. SALS localized to the pointed ends of growing thin filaments, but near the Z-line in mature muscle.^38^ Interestingly, SALS contains proline-rich profilin-binding sequences, which suggests that it may work with profilin to induce thin filament elongation from the pointed ends. Overexpression of SALS also promoted filament growth by potentially antagonizing Tmod capping activity.^38^ Since thin filament lengths are inversely proportional to the extent of Tmod-mediated capping,^39,40^ excessive profilin may help recruit SALS to the pointed ends of mature thin filaments, disrupt T-mod capping, and promote elongation via an ‘annealing mechanism’ as recently proposed for DAAM, a sarcomere-associated actin assembly factor, and a member of the formin family.^41^ Furthermore, high profilin levels are associated with disrupted myofilament packing, order, and integrity (*Figure 3D*, inset) and consequently impaired muscle function. Abnormalities and disarray in myofibrillar structure are also found in hypertrophic and failing hearts.^42^

### 4.3 Role of profilin-1 during hypertrophy

*Drosophila* has proved to be an efficient and effective model to study cardiomyopathy.^43^ Recent evidence reveals that deficits in conserved contractile components in flies induce pathological phenotypes remarkably similar to those that characterize human heart disease.^30,31,44^ A main advantage of this model is that it allows the role of profilin to be studied exclusively in the intact heart. Cardiomyocytes expressing elevated quantities of profilin resulted in cardiomyopathy characterized by increased cardiac dimensions, reminiscent of mammalian eccentric hypertrophy.^36^ This indicates that increased amounts of profilin are sufficient to induce a hypertrophic phenotype, which was confirmed in NRVMs, a widely accepted model for investigating cellular hypertrophy. To elucidate whether profilin-1 is also necessary for hypertrophy, we employed NRVMs in conjunction with *Pfn1* silencing. PE-induced hypertrophy of NRVMs was characterized by increased cell dimensions and fetal gene re-expression. This maladaptive hypertrophic response was significantly attenuated upon suppression of *Pfn1*. Up-regulation of fetal gene expression occurs as an early immediate response and was observed after 24 h of PE treatment. This indicates that profilin-1 is required, potentially during an initial phase at the onset of cellular remodelling, for cardiomyocyte hypertrophy.

Profilins perform a host of molecular roles by interacting with diverse partners throughout the cell;^3^ thus, we propose they mediate cardiac hypertrophy via numerous interrelated mechanisms. For example, elevated profilin-1 and altered thin filament and sarcomeric structure can directly affect the generation and propagation of contractile stress. Such mechanical changes are considered a trigger for cardiac remodelling by potentially modulating nodal signalling molecules throughout the myocyte cytoarchitecture.^45,46^ Profilin-1 may also stimulate common hypertrophic signalling cascades within cardiomyocytes including the MAPK pathway, consistent with our findings that reduced activation of ERK1/2, Raf, and transcription of the downstream genes *CTGF* and *IL-6* correlated with silencing of *Pfn1* translation. Similar results were obtained using vascular smooth muscle cells, which also showed ERK1/2-associated hypertrophy.^13,47^ Involvement of the MAPK pathway is consistent with multiple studies that have revealed the Ras/Raf/MEK1/ERK signalling pathway routinely promotes hypertrophy.^48^ While our data suggest that profilin-1 works upstream of Ras, we cannot conclude it activates ERK signalling through direct interactions with the small GTPases. However, several profilin ligands are well-known Rac and Rho effector molecules, which may assist in initiating the hypertrophic signal transduction pathway. Moreover, the hearts of transgenic mice overexpressing Gαq, which showed increased profilin abundance concomitant with hypertrophy, were previously characterized by reduced PIP2 levels that enhanced cardiomyocyte apoptosis and subsequent HF.^46^ Profilin-1 can bind PIP2 directly, and elevated levels may disproportionately deplete the phosphatidylinositol lipid, affect its availability for signal transduction, and contribute to cardiac remodelling. Finally, profilin can bind to and regulate the activity of the transcription factor p42*^POP^*, which is abundantly expressed in the heart.^11^ Elevated profilin levels may consequently markedly repress gene activity that directly or indirectly promotes cardiac remodelling. Overall, our study reveals complex functions of profilin as a modulator of sarcomeric organization and as a mediator of hypertrophic cardiomyocyte remodelling. Therefore, profilin-1 represents a potential therapeutic target to mitigate multiple aspects of hypertrophy directly, in both the myocardium and in the vasculature, during HF.

## Supplementary material

Supplementary material is available at *Cardiovascular Research* online.

## Funding

This work was supported by American Heart Association post-doctoral fellowship (V.K., 12POST11520006), British Heart Foundation Project Grant (V.K., PG/14/44/30890), National Institutes of Health [W.S., T-32 HL-07227; S.I.B., R01GM32443; J.V.E., P01 HL77189-01; J.V.E., NHLBI-HV-10-05 (2); and A.C., NHLBI R56HL124091 and R01HL124091], and American Heart Association scientist development grant (A.C., 10SDG4180089).
